# Self-injury from early adolescence to early adulthood: age-related course, recurrence, and services use in males and females from the community

**DOI:** 10.1007/s00787-020-01573-w

**Published:** 2020-06-22

**Authors:** Annekatrin Steinhoff, Denis Ribeaud, Stephan Kupferschmid, Nesrin Raible-Destan, Boris B. Quednow, Urs Hepp, Manuel Eisner, Lilly Shanahan

**Affiliations:** 1 Jacobs Center for Productive Youth Development, University of Zurich, Andreasstrasse 15, 8050 Zurich, Switzerland; 2 Integrated Psychiatric Services Winterthur, Zurcher Unterland, P. O. Box 144, 8408 Winterthur, Switzerland; 3 Experimental and Clinical Pharmacopsychology, Department of Psychiatry, Psychotherapy and Psychosomatics, Psychiatric Hospital of the University of Zurich, Lenggstrasse 31, PO Box 1931, 8032 Zurich, Switzerland; 4 Neuroscience Center Zurich, University of Zurich and Swiss Federal Institute of Technology, Winterthurerstr. 190, Y55 J04, 8057 Zurich, Switzerland; 5 Institute of Criminology, University of Cambridge, Sidgwick Avenue, Cambridge, CB3 9DA UK; 6 Department of Psychology, University of Zurich, Binzmühlestrasse 14, Box 1, 8050 Zurich, Switzerland

**Keywords:** Self-injury, Adolescence, Young adulthood, Services use, Sex differences, Longitudinal

## Abstract

Adolescent self-injury is a widespread public health problem, but long-term longitudinal studies from European countries are rare. Self-injury in males and sex differences are poorly understood. This study describes the prevalence, frequency, age-related course, and recurrence of, and mental health services use related to adolescent self-injury. Data came from a Swiss prospective-longitudinal cohort study (*N* = 1482). Adolescents (52% male) reported frequency of self-injury and mental health services use (including reasons for and types of services use, hospitalizations) at ages 13, 15, 17, and 20. Between ages 13–20, 27% of adolescents reported self-injury at least once. In males, prevalence decreased from 12 to 5%; in females self-injury peaked at age 15 (16%) and then decreased (11% at age 20). In males, recurrence of self-injury increased after age 15 (from odds ratio [OR] < 3 to OR > 10); in females, recurrence was high from age 13 onwards (OR > 5). Predictors of recurrence included childhood/early adolescent internalizing symptoms and early self-injury onset. Typically, less than half of adolescents with self-injury used mental health services. Males with self-injury used services mainly for externalizing problems, learning difficulties, and attention/concentration problems; females for depression or self-injury, family problems, and victimization. Types of services used changed with age, and adolescents with self-injury had increased rates of hospitalization. There are notable sex differences in the longitudinal course of self-injury and reasons for related mental health services use. Treating early internalizing symptoms could be a promising target for preventing recurrent self-injury. Males are at particular risk of not receiving adequate treatment for self-injury.

**Electronic supplementary material:**

The online version of this article (10.1007/s00787-020-01573-w) contains supplementary material, which is available to authorized users.

## Introduction

Self-harm, including self-injury (e.g., self-cutting), is a major public health problem among adolescents around the globe [1–3]. In light of recent rises in depressive symptoms, suicidality, and related mental health issues among adolescents, adolescent self-injury is receiving increased attention from researchers [4, 5]. However, several questions about adolescent self-injury have yet to be answered. First, how does the prevalence and frequency of self-injury unfold across adolescence in the community? Second, how recurrent is adolescent self-injury, and what predicts recurrence of self-injury across the adolescent years? Third, to what extent and for what reasons do adolescents with self-injury use mental health services? Finally, do males and females differ in their age-related course of and services use for self-injury? Answering these questions could provide valuable insights into when and how interventions to reduce the risk of self-injury among young people are most effective, and who such interventions should address. We obtained our data from a large community-representative cohort study with four repeated assessments of self-injury from early adolescence to early adulthood to address these questions.

### Definition of self-injury

Two traditions in research on self-injurious behaviors inform our conception of this behavior. One is research on self-harm (also termed “deliberate self-harm”), which is a broad term encompassing various behaviors that purposefully damage one’s own body, regardless of suicidal intent [6, 7]. The literature on self-harm typically includes direct self-injurious behaviors, such as cutting, as well as other detrimental behaviors, such as self-poisoning [1, 7–9]. Another line of research is devoted specifically to self-harming behaviors pursued without suicidal intent, termed non-suicidal self-injury (NSSI) [10, 11]; prototypical behaviors addressed in this line of research include cutting, scratching, and interfering with wound healing. The current study focuses on the latter forms of self-injurious behaviors but does not distinguish between behaviors based on the presence or absence of suicidal intent. Therefore, both the self-harm and NSSI research traditions are relevant here.

### Age-related course and recurrence of self-injury

Lifetime prevalence rates of self-harm and NSSI in community samples of adolescents are typically 16–18% [10]. Shorter-term longitudinal studies suggest that self-injurious behaviors emerge in early adolescence (~ age 13), increase and peak in mid-adolescence (~ ages 15–17), and then decrease [12].

Documentation of the age-related course of self-injury is currently fragmented in both the line of research on NSSI and that on the broader self-harm framework. This is due to a lack of community-based studies that repeatedly assess the same individuals from early adolescence (i.e., when self-harm and NSSI typically emerge) into early adulthood [12]. Furthermore, few of the existing longitudinal studies are based on European data [13]. Extant longitudinal data on self-injury also tend to over-represent females [14–17]; therefore, knowledge about male self-injury is limited. Although some studies suggest that the risk of self-injury is higher among females than males [18] and that methods of self-injury vary according to sex [19], the literature is inconclusive about how sex differences in self-injury develop during adolescence. Adolescent depression—which has risen in the past decade [5] and is comorbid with self-injury in a significant group of young people [20]—follows a dramatically sex-differentiated course, with female adolescents exhibiting more depressive symptoms than males, particularly during early and mid-adolescence [21]. It is not known whether adolescent self-injury mirrors this sex-differentiated age-related course. One cross-sectional study suggests that sex differences in self-injury emerge only after early adolescence [22]. However, it has yet to be confirmed whether this finding can be replicated with longitudinal panel data.

With regard to the recurrence of self-injury (i.e., habitualization leading to repeated self-injury over prolonged periods), theories suggest that youth who self-injure are likely to adopt this behavior as their primary stress relief strategy [23], although this has not yet been tested empirically using prospective longitudinal data from a community sample. Self-injury is thought to be a moderately stable behavior [24], but we do not know whether patterns of recurrence change with increasing age, whether recurrence differs by sex, and what sociodemographic factors, aspects of self-injury (e.g., age of onset, frequency), and associated mental health problems (e.g., depressive symptoms, suicidal ideation) predict recurrence of self-injury between early adolescence and early adulthood. It is well known that childhood adversity predicts adolescent self-injury, and some of these effects could be mediated by depressive and anxiety symptoms [25, 26]. However, prospective studies on whether childhood internalizing symptoms increase the risk of recurrence of self-injury over prolonged periods of adolescence and until early adulthood are currently lacking.

### Mental health services use among adolescents with self-injury

Self-injury may require psychological, psychiatric, or general medical in- or outpatient services. Indeed, adolescent self-harm is associated with increased emergency department use and inpatient admissions [27]. Research on services use suggests that females are typically more willing than males to use mental health services [28]. However, little is known about the types of services used, the reasons for services use, or hospitalizations in the context of adolescent self-injury in the community. We examined whether adolescents who self-injured (1) used school-based or clinical mental health services and for what reasons and (2) were admitted to a psychiatric or a general hospital. We also examined sex differences in services use.

## Methods

### Sampling and procedures

Data came from the prospective longitudinal Zurich Project on the Social Development from Childhood to Adulthood (z-proso) [29]. Participants were selected using a cluster-stratified randomized sampling approach. A total of 1675 children from 56 primary schools were randomly selected from 90 public schools in Zurich, Switzerland’s largest city. The stratification took into account school size and socio-economic background of the school district. Participants were first assessed in 2004 and were largely representative of first graders attending public schools in Zurich. Participants were assessed seven more times since then (most recently in 2018). The mean ages at the assessment points used in our study were 7.45 (SD 0.38), 11.33 (SD 0.37), 13.67 (SD 0.36), 15.44 (SD 0.36), 17.45 (SD 0.37), and 20.58 (SD 0.38). In what follows, we refer to the different waves by rounded mean ages (i.e., 7, 11, 13, 15, 17, and 20).

Data were collected through computer-assisted interviews in the first three waves (ages 7–9) and paper-and-pencil questionnaires at ages 11, 13, 15, and 17. At age 20, participants completed their surveys on a computer at a university research laboratory. Interviews at ages 7–9 lasted approximately 45 min; at ages 11–20, they lasted about 90 min. Parents and teachers were also assessed during the first waves of data collection. Adolescents received a cash incentive for their participation, increasing from ~ $30 at age 13 to ~ $75 at age 20. Topics addressed during the interviews included various behavioral, emotional, and social experiences. Self-injury was assessed at ages 13–20 (i.e., during the last four assessments only).

The study was conducted in accordance with national and international ethical standards and was approved by the responsible ethics committee at the Faculty of Arts and Social Sciences, University of Zurich. Adolescents provided written informed consent at each wave; until age 15 parents could opt their child out of the study.

### Participants

A total of *N* = 1482 adolescents participated at least once between ages 13 and 20, when self-injury was assessed (52% male, 91% born in Switzerland). In 26% of households, ≥ 1 parent held a university degree. Participants’ household occupational status scores on the International Socio-Economic Index [30]—calculated using occupation-specific income and the required educational level—averaged 45.74 (SD 19.24), range 16–90 (i.e., from unskilled worker to judge); 76% of adolescents (*n* = 1104/1445) had ≥ 1 immigrant parent, which is consistent with the city’s parent population composition (see Online Supplement A).

### Measures

*Self-injury* was assessed at ages 13, 15, 17, and 20 with one item that asked how often adolescents had self-injured on purpose during the past month. Several example behaviors were provided [“I harmed myself on purpose (e.g., cut my arm, tore wounds open, hit my head, tore out my hair)”]. This broad assessment focused on direct self-injurious behaviors and did not include self-poisoning (e.g., overdosing on a drug or swallowing chemicals). It also did not distinguish particular motivations or severity of the injury and could, therefore, include both suicidal self-injury and NSSI. Answers were recorded on a five-point scale (1 = never, 2 = rarely, 3 = sometimes, 4 = often, 5 = very often). We used the full frequency scale and also created a dichotomized variable to assess prevalence (0 = never, 1 = rarely or more, the latter indicating once or more in the past month). Furthermore, we created a binary variable that distinguished between frequent (categories 4 and 5) vs. less frequent (categories 2 and 3) self-injury. Online Supplement B shows significant positive associations between self-injury and internalizing symptoms and between self-injury and suicidal ideation in our sample, which is consistent with findings from other studies and supports the validity of our assessment [20, 31].

*Mental health services use* was assessed at ages 13, 15, 17, and 20. Adolescents reported whether they had seen a school-based provider (i.e., a school psychologist, school social worker, or school-based psychosocial counselor or counseling center) or clinician (i.e., a clinical psychologist or psychiatrist) since the last assessment (typically the past 2 years, at age 20 the past 3 years). These categories of services constitute the major mental health services avenues for adolescents in Switzerland. Adolescents also reported reasons for services use, selecting ≥ 1 possible mental health reasons (i.e., “depression/self-injury/suicidal thoughts”, “learning difficulties”, “attention deficit/difficulties with concentrating”, “drug/alcohol use”), social reasons (i.e., “family problems”, “violence/bullying involvement as perpetrator”, “victimization”, “problems with teachers”), or “other” reasons.

*Hospitalization* was measured from age 13 to 20. Adolescents indicated whether they had been admitted to any hospital for several days since the last assessment; at ages 17 and 20, inpatient psychiatric hospital admission was also assessed.

*Internalizing symptoms* during the past month were assessed at ages 11, 13, 15, 17, and 20 using eight items from the Social Behavior Questionnaire concerning depressive and anxiety symptoms [32]. Assessments were made on a five-point scale (1 = never to 5 = very often). The internal consistency of the scale was adequate (Cronbach’s *α* = 0.79, 0.82, 0.84, 0.85, 0.87 at ages 11, 13, 15, 17, and 20, respectively).

At ages 15, 17, and 20, respondents also reported how often during the past month they had thought about suicide using a 5-point scale (1 = never to 5 = very often). We used a dichotomized variable, with 1 = any suicidal thoughts and 0 = no suicidal thoughts. Suicide attempts were not assessed.

*Parental income* was assessed in the first wave of the survey (assessment at age 7). Parents reported their household income on a 10-point scale ranging from 1 = 0–1999 CHF/month to 10 ≥ 15,000 CHF/month (*M* = 6.02, SD 1.95).

*Parental education* indicates whether at least one parent held a university degree.

*Migration background* indicates whether both parents were born abroad (vs. at least one parent born in Switzerland).

### Analytic strategy

Point prevalence estimates of self-injury included participants with valid data on self-injury at each assessment (*N* = 1362; *N* = 1443; *N* = 1305; *N* = 1180 at ages 13, 15, 17, and 20, respectively; a total of 1482 respondents participated and provided data on self-injury at least once between ages 13 and 20). Cumulative prevalence estimates of self-injury were derived by aggregating each adolescent’s data from age 13 to 20; these assessments included participants with valid data at all assessments only (*N* = 1030). The shape of the age-related course of self-injury was modeled with generalized estimating equations, which are useful for providing robust standard errors for study designs with within-person repeated measurements. Longitudinal recurrence of self-injury and sex differences were calculated using binary logistic regression models. In the models examining recurrence of self-injury, the presence or absence of self-injury at a given wave was the dependent variable and self-injury at the previous wave was the predictor. These models also included interaction terms (i.e., sex*prior self-injury) to test sex differences in the recurrence of self-injury. Predictors of self-injury recurrence were analyzed using multinomial logistic regression models. We analyzed predictors of any recurrence between ages 13 and 20 (vs. one-time self-injury and no self-injury) and of wave-to-wave recurrence. For the latter analyses, respondents were assigned to one of three groups: those with recurrent self-injury [i.e., self-injury at consecutive assessments (13 and 15, 15 and 17, or 17 and 20, respectively)], those with self-injury at the respective first assessment only (i.e., at age 13 for the period from age 13 to 15, at age 15 for the period from age 15 to 17, or at age 17 for the period from age 17 to 20), and those without any self-injury at the respective first assessment.

In the recurrence analyses, we applied multiple imputation to missing data. This technique reduces the potential bias that can follow from selective attrition mechanisms, which are common in longitudinal research [33, 34]. To analyze attrition mechanisms, we compared age 13 respondents who still participated at age 20 to those who did not participate at age 20. Self-injury at age 13 was not related to study participation at age 20 (*p* = 0.53). However, male adolescents (*p* = 0.000), adolescents whose parents did not hold a university degree (*p* = 0.000), and adolescents whose parents were both born abroad (*p* = 0.002) were less likely than their peers to participate in the survey at age 20. Parental income was higher among those who still participated at age 20 (*p* = 0.000). Respondents who did not participate at age 20 had fewer internalizing symptoms at age 13 than those who still participated at age 20 (*p* = 0.000).

Analyses of prevalence and age-related course of self-injury and respective sex differences were conducted in SPSS [35]. Multiple imputation and the models for analyzing predictors of recurrent self-injury were specified in Mplus [36].

## Results

### Prevalence, frequency, and course of self-injury

#### Overall

**Fig. 1:**
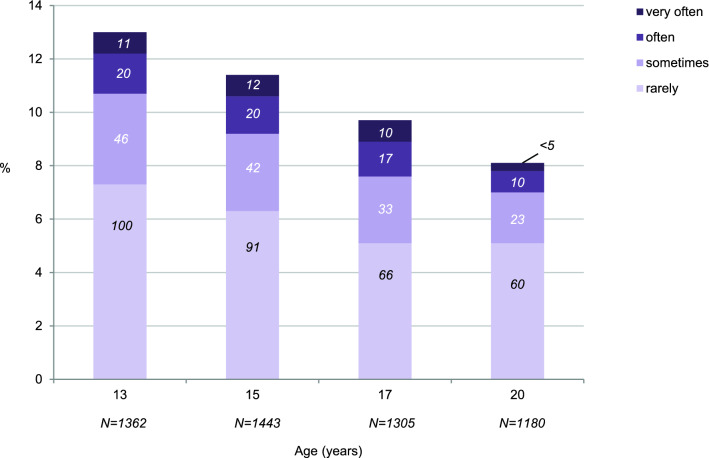
Point prevalence and frequency of self-injury from age 13 to 20 (numbers in bars indicate *n*)

About one in four adolescents (27%, *n* = 278) reported self-injury at least once between ages 13 and 20. Point prevalence (Fig. 1) was highest at age 13 and subsequently decreased (odds ratio [OR] 0.95, 95% CI 0.93–0.97, *p* < 0.001 for age-related decrease). Most adolescents with self-injury reported a frequency of “rarely” or “sometimes” in the past month; a minority self-injured “often” or “very often” (> 2% of the entire sample at age 13–17; > 1% of the entire sample at age 20).

#### By sex

**Fig. 2:**
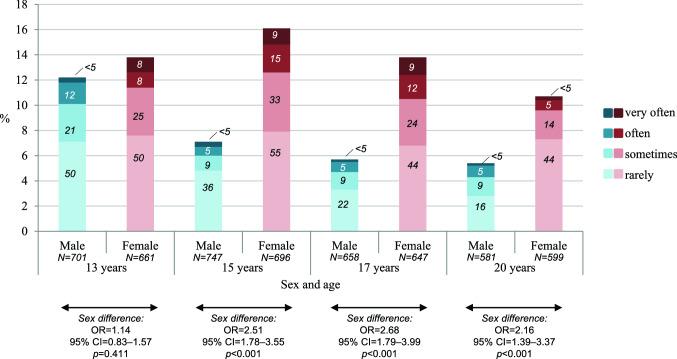
Sex-specific prevalence and frequency of self-injury from age 13 to 20 (numbers in bars indicate *n*)

One in three females (32%, *n* = 170) and one in five males (21%, *n* = 108) self-injured at least once between ages 13 and 20 (OR 1.76, 95% CI 1.33–2.33, *p* < 0.001 for sex difference). The age-related course of self-injury was sex-differentiated. Beginning at age 15, females’ point prevalence was higher than males’ (see Fig. 2 for prevalence estimates and OR). Indeed, females’ self-injury peaked at age 15—when one in six females reported it—and then declined. In contrast, males’ self-injury was highest at age 13 and then declined. Regressing self-injury on age and age-squared (using a continuous variable to code respondents’ exact ages) revealed a significant curvilinear pattern in females (OR(age^2^) 0.99, 95% CI 0.98– < 1.00, *p* = 0.015). A similar pattern but of the opposite shape was observed in males (OR(age^2^) 1.03, 95% CI 1.02–1.04, *p* < 0.001; i.e., the prevalence of self-injury decelerated steeply between ages 13 and 15 and then more slowly). These findings support the notion that self-injury peaks at different ages in male and female adolescents. Among youth with self-injury, there were no significant sex differences in the frequency of the behavior (i.e., rarely/sometimes vs. often/very often: OR 1.01, 95% CI 0.47–2.19, *p* = 0.98 at age 13; OR 1.53, 95% CI 0.64–3.69, *p* = 0.34 at age 15; OR 1.60, 95% CI 0.59–4.35, *p* = 0.36 at age 17; OR 0.50, 95% CI 0.15–1.65, *p* = 0.26 at age 20).

### Recurrence of self-injury

#### Overall

**Fig. 3:**
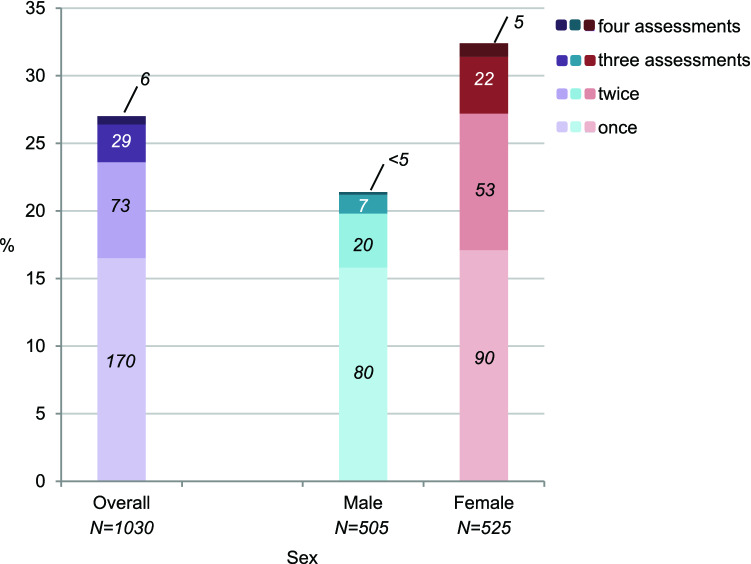
Number of assessments during which adolescents reported any self-injury (numbers in bars indicate *n*)

Self-injury at the previous assessment predicted current self-injury (OR 4.35, 95% CI 2.94–6.43, *p* < 0.001 for age 13→15; OR 8.45, 95% CI 5.59–12.77, *p* < 0.001 for age 15→17; OR 10.46, 95% CI 6.48–16.88, *p* < 0.001 for age 17→20). Notably, increases in the recurrence of self-injury with age were significant (*p* = 0.005). In the self-injury group, most adolescents reported self-injury only once during the assessment period (Fig. 3; 17% of overall sample). Nevertheless, almost one in three adolescents in the self-injury group reported the behavior during at least two assessments (11% of sample).

#### By sex

In females, self-injury was highly recurrent from age 13 onward (OR 5.91, 95% CI 3.58–9.75, *p* < 0.001 for age 13→15; OR 6.42, 95% CI 3.91–10.52, *p* < 0.001 for age 15→17; OR 7.39, 95% CI 4.17–13.11, *p* < 0.001 for age 17→20). In males, recurrence was lower between ages 13–15 but then increased (OR 2.64, 95% CI 1.31–5.31, *p* = 0.006 for age 13→15; OR 10.51, 95% CI 4.87–22.66, *p* < 0.001 for age 15→17; OR 17.03, 95% CI 7.02–41.30, *p* < 0.001 for age 17→20; *p-*values for sex difference in recurrence were 0.066, 0.29, and 0.12 for 13→15, 15→17, and 17→20, respectively). Almost half of the females in the self-injury group reported the behavior during at least two assessments (Fig. 3; 15% of females in sample), compared to one in four males in the self-injury group (6% of males in sample; OR 2.54, 95% CI 1.50–4.29, *p* < 0.001 for sex difference).

### Predictors of recurrence

Recurrence of self-injury was independent of parental education, income, and migration background. The results presented in Table 1 provide a characterization of youth with one-time vs. recurrent self-injury. Predictors of recurrence included being female, higher internalizing symptoms in early adolescence, early-adolescent onset of self-injury (particularly among females), and frequent self-injury during early adolescence.

**Table 1:** Predicting recurrence of self-injury: results from multinomial logistic regression models, OR (95% CI); all main predictors were tested separately and adjusted for parental education, income, migration background, and sex (*N* = 1482)

	One-time and recurrent self-injury between ages 13 and 20			Wave-to-wave recurrence of self-injury		
Predictors	One-time self-injury vs. no self-injury	Recurrent self-injury vs. no self-injury	Recurrent self-injury vs. one-time self-injury	Recurrent self-injury at ages 13 and 15 vs. self-injury at age 13 only^d^	Recurrent self-injury at ages 15 and 17 vs. self-injury at age 15 only^d^	Recurrent self-injury at ages 17 and 20 vs. self-injury at age 17 only^d^
Parental education	0.81 (0.54–1.21)	0.77 (0.47–1.26)	0.95 (0.53–1.71)	0.55 (0.22–1.42)	1.72 (0.67–4.40)	1.33 (0.47–3.81)
Parental income	0.94 (0.85–1.04)	0.94 (0.84–1.06)	1.00 (0.88–1.15)	1.08 (0.89–1.32)	0.97 (0.79–1.20)	0.96 (0.74–1.24)
Parental migration background	1.04 (0.77–1.41)	0.72 (0.50–1.03)	0.69 (0.44–1.06)	0.49 (0.25–0.95)	0.86 (0.44–1.68)	0.97 (0.43–2.20)
Female sex	1.24 (0.93–1.66)	**2.92** (1.98–4.29)	**2.36** (1.49–3.73)	**3.92** (1.93–7.96)	1.53 (0.74–3.18)	0.95 (0.39–2.29)
Main predictors						
Childhood internalizing symptoms (age 11)	**1.39** (1.09–1.78)	**1.94** (1.45–2.59)	1.39 (1.00–1.94)	1.49 (0.93–2.38)	**1.68** (1.01–2.81)	**2.20** (1.28–3.79)
Early adolescent internalizing symptoms (age 13)	**2.26** (1.82–2.81)	**3.50** (2.69–4.55)	**1.55** (1.17–2.06)	**2.16** (1.43–3.26)	**1.62** (1.08–2.42)	1.33 (0.82–2.16)
Internalizing symptoms at baseline^a^ (if not age 13)	–	**–**	–	–	**2.43** (1.54–3.84)	1.06 (0.68–1.65)
Early adolescent onset of self-injury (age 13)	–	**–**	**2.05** (1.26–3.33)	–	1.87 (0.89–3.95)	0.89 (0.39–2.04)
Early adolescent onset of frequent self-injury^b^ (age 13)	–	**–**	**2.55** (1.10–5.90)	2.47 (0.89–6.83)	**3.28** (1.04–10.38)	1.26 (0.31–5.13)
Frequent self-injury at baseline (if not age 13)^b^	–	**–**	**–**	**–**	1.70 (0.73–3.96)	0.85 (0.34–2.17)
Suicidal ideations at baseline^a,c^	–	–	–	–	**2.06** (1.02–4.15)	1.49 (0.61–3.68)

Bold value indicates significant results (p < 0.05)

^a^Baseline refers to the first assessment of the respective period (i.e., age 13 for recurrent self-injury at ages 13–15, age 15 for recurrent self-injury at ages 15 to 17, and age 17 for recurrent self-injury at ages 17–20)

^b^Binary coding: often/very often vs. rarely/sometimes/none

^c^Suicidal ideations were first assessed at age 15 and were only included as predictors when assessed prior to the outcome

^d^Multinomial regression analyses included comparisons between three groups: those with recurrent self-injury [i.e., self-injury at consecutive assessments (13 and 15, 15 and 17, or 17 and 20, respectively)], those with self-injury at the respective first assessment (i.e., baseline) only (i.e., at age 13 for the period from age 13 to 15, at age 15 for the period from age 15 to 17, or at age 17 for the period from age 17 to 20), and those without any self-injury at the respective first assessment (i.e., baseline). Comparisons between those with recurrent self-injury and those with no self-injury at baseline and between those with self-injury at baseline only and those without self-injury at baseline are not displayed

With respect to wave-to-wave recurrence, 13-year-olds were at increased risk of continued self-injury until age 15 if they were female or had comorbid internalizing symptoms at age 13. Fifteen-year-olds were at increased risk of continued self-injury until age 17 if they had initiated frequent self-injurious behavior early (i.e., reported at age 13), had a history of internalizing symptoms across late childhood and early to mid-adolescence, or reported suicidal ideations at age 15. Finally, 17-year-olds were at increased risk of continued self-injury until age 20 if they had a history of childhood internalizing symptoms. There were no significant interactions between sex and any of the variables listed in Table 1 in predicting wave-to-wave recurrence of self-injury.

### Services use among adolescents with self-injury

#### Overall mental health services use

From age 13 to 20, adolescents with self-injury reported more use of services than those without self-injury (75% [204/273] vs. 59% [442/744]; OR 2.02, 95% CI 1.48–2.75, *p* < 0.001 for group difference), including clinical services (OR 2.42, 95% CI 1.82–3.21, *p* < 0.001) and school-based services or counseling (OR 1.75, 95% CI 1.32–2.33, *p* < 0.001). Youth with recurrent self-injury did not differ significantly from those with one-time self-injury in their overall mental health services use (77%, [82/106] vs. 73% [122/167]; OR 1.26, 95% CI 0.71–2.23, *p* = 0.43), but recurrent self-injury was associated with more clinical services use (62% [66/107] vs. 44%, [73/167]; OR 2.07, 95% CI 1.26–3.40, *p* = 0.004). Notably, the point prevalence estimates (see Online Supplement C) show that services use among adolescents with self-injury was < 50% at most assessments. The age-related course of services use presented in the supplement shows that there was an overall increase of services use from early adolescence to early adulthood, which was mainly due to an increasing number of respondents with self-injury using clinical services. The use of school-based services declined with age.

#### Overall hospitalizations

Forty-three percent of adolescents with self-injury (119/277) had been admitted to a general hospital at least once between ages 13 and 20, compared to 36% of those without self-injury (265/746; OR 1.37, 95% CI 1.03–1.81, *p* = 0.029). In addition, 22% of adolescents with self-injury between ages 17 and 20 (35/163) were admitted to a psychiatric hospital. This compared to 3% of those without self-injury having psychiatric hospitalizations (28/951; OR 9.01, 95% CI 5.30–15.32, *p* < 0.001).

#### By sex

Seventy-eight percent (132/169) of females and 69% (72/104) of males with self-injury had used mental health services (non-significant sex difference) at least once between ages 13 and 20, and 26% (30/116) of females and 11% (5/47) of males with self-injury between ages 17 and 20 had been admitted to a psychiatric hospital (OR 2.93, 95% CI 1.06–8.10, *p* = 0.038 for sex difference). No sex difference was observed for admission to a general hospital. Online Supplement C shows the sex-specific point prevalence of services use during the past 2 or 3 years.

### Reasons for services use

#### Overall

**Fig. 4:**
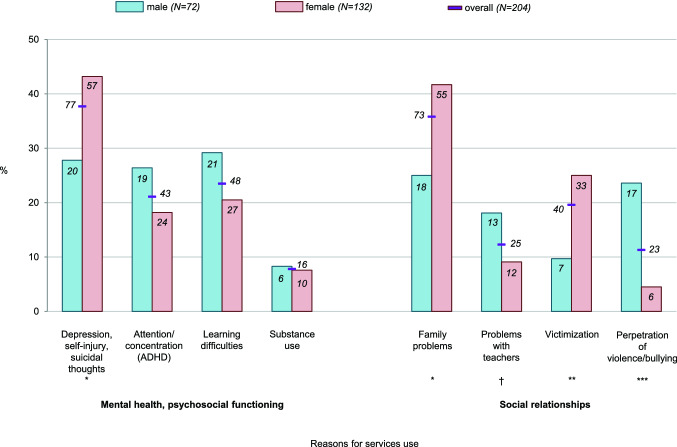
Overall and sex-specific cumulative prevalence of reasons for services use among adolescents with any self-injury at least once between ages 13 and 20 (numbers in bars/next to the lines indicate *n*). ^†^*p* < 0.10, *p < 0.05, **p < 0.01, ***p < 0.001

One in three adolescents with self-injury who used services reported depression, self-injury, or suicidal thoughts as a reason for services use between ages 13 and 20 (Fig. 4; see also Online Supplement B for significant associations between self-injury and depressive symptoms/suicidal ideation in our sample). However, these reasons were assessed with one survey question only and could not be further disaggregated. The next most common mental health reasons for services use by adolescents with self-injury were attention or concentration problems, learning difficulties, and substance use. With respect to social reasons, family problems were most common, followed by victimization, problems with teachers, and perpetration of violence or bullying.

Adolescents with self-injury indicated depression/self-injury/suicidal thoughts, substance use, family problems, and victimization as reasons for services use more often than adolescents without self-injury who had used services (Table 2). Adolescents with recurrent self-injury were more likely than those with one-time self-injury to have used services due to depression, self-injury, or suicidal thoughts (55% [45/82] vs. 26%, [32/122], OR 3.42, 95% CI 1.89–6.19, *p* < 0.001). Online Supplement C provides the point prevalence of reasons for services use.

**Table 2:** Differences in reasons for services use among adolescents with and without self-injury and sex differences within the groups

Reasons	Adolescents with vs. without self-injury			Adolescents with self-injury: female vs. male			Adolescents without self-injury: female vs. male		
	OR	95% CI	*p*	OR	95% CI	*p*	OR	95% CI	*p*
Mental health/psychosocial functioning									
Depression, self-injury, suicidal thoughts	4.65	3.10–6.98	< 0.001	1.98	1.06–3.67	0.031	1.55	0.86–2.78	0.15
Attention/concentration problems (ADHD)	1.03	0.69–1.55	0.89	0.62	0.31–1.23	0.17	0.39	0.24–0.64	0.39
Learning difficulties	1.29	0.87–1.93	0.87	0.62	0.32–1.21	0.16	0.78	0.49–1.26	0.31
Substance use	2.13	1.05–4.30	0.036	0.90	0.31–2.59	0.85	0.33	0.11–1.03	0.056
Social relationships									
Family problems	1.86	1.29–2.67	< 0.001	2.14	1.14–4.05	0.019	2.27	1.44–3.57	< 0.001
Problems with teacher	1.30	0.77–2.19	0.33	0.45	0.20–1.06	0.067	0.87	0.46–1.64	0.67
Victimization	1.91	1.21–3.01	0.005	3.10	1.29–7.41	0.011	1.48	0.82–2.68	0.19
Perpetration of violence or bullying	0.88	0.52–1.47	0.62	0.15	0.06–0.41	< 0.001	0.53	0.29–0.95	0.034

#### By sex

Females with self-injury were more likely than males with self-injury to use mental health services for depression, self-injury, or suicidal thoughts at least once between ages 13 and 20 (Fig. 4; Table 2); no sex difference with respect to this reason emerged among adolescents without self-injury. Females with self-injury were more likely to use services due to family problems and victimization than males. In contrast, males were more likely to use services because of problems with teachers, violence or bullying perpetration, or, in early adolescence, attention deficit or concentration problems or learning difficulties (see Online Supplement C for details on the point prevalence of reasons for services use by sex).

## Discussion

It has become a widespread phenomenon that adolescents, who are on the brink of tackling important developmental milestones, purposefully inflict physical harm upon themselves, potentially with long-term negative consequences [37]. Ours is among the first studies to examine self-injury using longitudinal community data on early adolescence to young adulthood to illuminate the age-related course and recurrence of self-injury and the use of mental health services among adolescents with self-injury. Furthermore, this study generates important knowledge about sex differences in the age-related course of and services use for self-injury across adolescence. Our findings show that sex differences in the prevalence of self-injury emerge only in mid-adolescence (not earlier) and that recurrence of self-injury increases with age, particularly among males. Internalizing symptoms in late childhood and early adolescence increase the risk of recurrent self-injury among both males and females and deserve special attention for the prevention of adolescent and early-adult self-injury. We found that less than half of adolescents with self-injury had used mental health services at most assessment points.

### High prevalence of self-injury

Our cumulative prevalence estimate for self-injury was 27%, which falls within the higher range of previous estimates [2, 38]. This could be due to several reasons. First, prior studies did not assess self-injury prospectively over prolonged periods, including in early adolescence, which is when the behavior typically emerges [12]. Measuring cumulative prevalence across four assessments is a more reliable assessment than single-time retrospective reports, which tend to result in lower prevalence estimates due to forgetting [39]. Second, the self-injury item used here listed a range of minor (e.g., hair-pulling) and major (e.g., cutting) example behaviors. This wide range of example behaviors likely resulted in higher estimates than narrower examples—which were often used in previous studies—would have yielded [19]. Third, the school system of the canton of Zurich is highly competitive during adolescence. Adolescents are academically monitored and placed into vocational school and college-bound tracks based on their grades and high-stakes testing at ages 12/13 and 15/16. Such academic performance pressures could increase the risk of self-injury, which has been observed in other countries [40]. Fourth, many adolescents in this sample had parents with a migration background, and although migration status per se was not related to (recurrence of) self-injury in our study, migration-related stressors or trauma (which were not assessed here) of some migrant parents could affect adolescents and increase the risk of distress and self-injury among this group. Overall, however, the high rates of self-injury in our sample correspond to high rates of suicidality among Swiss adolescents [41]. And, with the inclusion of the last assessment from 2018, our study may capture recent population-level increases in adolescent suicidal and self-injurious behaviors [4].

### Age-related course of self-injury from early adolescence to early adulthood

Declines in rates of self-injury from age 13 to 20 in the overall sample contrast with the findings of most previous work, which suggested a peak in mid-adolescence [12]. However, that work was primarily based on females. In our female-only analyses, we, too, found that self-injury prevalence peaked at age 15. It was the inclusion of males that contributed to linear age-related declines in self-injury in the overall sample.

The sex-differentiated course of self-injury prevalence could, in part, reflect sex-differentiated stress reactivity and other biological changes during mid-adolescence, which also contribute to the preponderance of depression among females at that age [21, 42–44]. Furthermore, in recent years, symptoms of depression are increasing in adolescent females in Western countries such as the US [5]. The high rates of female self-injury in our sample could be part of this new trend of increased emotional distress in adolescent females in Western societies.

Steep decreases in the prevalence of self-injury among males after age 13 are consistent with findings from a previous cross-sectional study comparing different age groups [22]. Nevertheless, one in five males reported self-injury at least once between early adolescence and young adulthood. This highlights the fact that self-injury among males is a serious concern and an urgent topic for research. The steep decreases after age 13 raise questions about how mid- and late-adolescent males cope with distress. Males who have previously self-injured could be adopting more adaptive coping strategies. However, it is more likely that they resort to other maladaptive “coping” strategies that are consistent with stereotypes of male-typical behavior (e.g., substance use, aggression, other risky behaviors). Indeed, many males in our sample (including those without self-injury) reported perpetration of violence and bullying as a primary reason for mental health services use.

During all assessments between ages 13 and 17, at least one in five youth who reported self-injury (i.e., > 2% of the overall sample) self-injured frequently (i.e., often or very often in the past month), which could indicate progression toward non-suicidal self-injury-disorder. This disorder was proposed in the recent Diagnostic and Statistical Manual of Mental Disorders (DSM-5) and includes “at least five instances of non-suicidal self-injury during the past year” as a diagnostic criterion [45]. Frequent self-injury that lasts into early adulthood could also be a symptom of borderline personality disorder.

### Recurrence of self-injury

More than one in ten adolescents from our sample reported recurrent self-injury and, consistent with previous theories suggesting that prior self-injury increases the risk of future self-injury, our findings revealed increasing wave-to-wave recurrence of self-injury from early to late adolescence [23, 46]. Early adolescents may be experimenting more with self-injury, perhaps due to social exposure to such behavior. In contrast, mid- and late adolescents could be adopting self-injury as a habitual coping or stress relief strategy. Increasing recurrence is consistent with the “implicit identification hypothesis,” according to which identifying with self-injury and valuing it as an effective coping strategy fosters its recurrence [23]. Early onset of self-injury, particularly of frequent self-injury, may be a gateway to a prolonged lifetime history of self-injury and, therefore, warrants attention from researchers and practitioners [see also, 47].

Our findings suggest that identification with self-injury and use of self-injury as a general stress relief strategy could be occurring at younger ages in females compared to males. The initial lower recurrence in males could reflect more sporadic and context-specific use of self-injury (e.g., when facing learning difficulties). A significant proportion of males who engage in self-injury during early adolescence may have subsequently used more “male-typical” stress relief strategies. Males who continued with self-injury past age 15 could have a high biological vulnerability to experiencing stress relief or reward through self-injury, which would explain its high recurrence among those who continued [for a more socialization theoretical perspective, see also 48].

Prior or concurrent internalizing symptomatology was associated with a substantially increased risk of self-injury recurrence over several years. This finding supports theories maintaining that internalizing symptoms could serve as a mediator between childhood adversity and self-injury in adulthood [25, 26]. Although we did not assess the potential starting point for this chain of events (i.e., childhood adversity), our study adds to prior evidence by providing prospective data indicating a link between internalizing symptoms in childhood and early adolescence and self-injury recurrence. Children with internalizing symptoms who discover self-injury as a way to relieve stress during their adolescent years may be at highest risk of not learning other, more adaptive, ways of coping with negative emotions, even as they transition into adulthood. Future research should further investigate how internalizing symptoms in childhood and early adolescence interact with adolescent self-injury in increasing the risk of self-injury habitualization.

Our findings suggest that, for females, the prevention of self-injury recurrence needs to begin in or before early adolescence. For males, prevention efforts could still be effective in early to mid-adolescence but would likely need to include strategies aimed at preventing alternative maladaptive coping strategies. However, although the prevalence of recurrent self-injury partially differed according to sex, associations between risk factors and recurrence of self-injury were not sex-specific (although the low prevalence of recurrent self-injury among males may have limited the statistical power to detect such differences). When working with adolescents with a history of self-injury, anamnesis of comorbid or prior depressive symptoms could be used to assess the risk of self-injury recurrence among both males and females. Tailoring psychotherapy to reduce the risk of recurrence could be one way to prevent long-term individual histories of self-injury.

### Prevalence of mental health services use and hospitalization in the contexts of self-injury

Self-injury was associated with an increased likelihood of mental health services use and of being admitted to a psychiatric hospital. Use of clinical services was particularly prevalent among adolescents with recurrent self-injury. Adolescents with self-injury were also more likely to be admitted to general hospitals, perhaps for injuries inflicted by self-injury and associated risky behaviors [2]. The point prevalence of services use remained below 50% at most ages (except age 20, when it was < 60%). This indicates underuse of mental health services. Switzerland offers universal health care, but our finding of underutilization of services is consistent with studies from countries without universal health care [49].

This raises the question of why many adolescents who self-injure do not use services. Potential reasons include low mental health literacy among adolescents and their parents, a lack of knowledge about available and adequate services, high thresholds for services use (e.g., long waiting times or financial costs when particular services are not covered by basic health insurance [50]), and (perceived) negative stigma and other consequences associated with seeking mental health services. This is particularly salient in adolescent males [28]. Adolescents could also fear loss of autonomy and disconnection from their home and community in the event of being hospitalized [51]. In addition, the supply of specialized child and adolescent psychiatric services is limited [50]. Finally, many adolescents engage in self-injury out of curiosity only and on a one-time or time-limited experimental basis. These adolescents may not require mental health services.

Notably, the primary source of services for early and mid-adolescents with self-injury were school-based services. Clinical services were used increasingly from late adolescence onward. The increase in (clinical) services use until young adulthood could be due to increasing severity of self-injurious behaviors with increasing age (e.g., cutting becomes more prevalent among older adolescents and young adults [22]). Increasing services use could also be indicative of increasing mental health literacy and self-responsibility with age [52]. The underutilization of services among the youngest adolescents is particularly concerning because of the high risk of recurrence and long-term psychiatric impairments that can follow from early self-injury [47, 53]. Our findings highlight a particular need to improve young adolescents’ and their parents’ knowledge about, and access to, professional interventions that have the potential to reduce both self-injury and associated behavioral and developmental risks. Such interventions could include intensive community care, cognitive behavioral therapy, and, particularly, dialectical behavioral therapy [54–57]. The latter focuses on skills training and the improvement of emotion regulation, acceptance, and distress tolerance [56, 57]. Low-threshold points of entry into services use at school may be key for successfully reaching adolescents in distress and could also be an important gateway into more specialized clinical services use [58].

### Reasons for mental health services use among adolescents with self-injury

Our study reveals sex differences in the reasons for services use among adolescents with self-injury. Females who self-injured frequently endorsed depression, self-injury, and suicidal thoughts as their reasons for services use, followed by family problems and peer victimization. These findings are consistent with female adolescents using self-injury as a coping strategy to alleviate distress [23].

In contrast, externalizing problems and, in early adolescence, learning difficulties and attention/concentration problems were typical reasons for mental health services use among males who self-injured. This could be consistent with the use of self-injury as a form of self-punishment or for social signaling purposes (e.g., as an expression of anger or aggression) [23, 59]. Indeed, more than 70% of males with self-injury received no services or received services primarily for problems other than self-injury, which means that their self-injury may not have been adequately treated (possibly resulting in higher recurrence by mid-adolescence) [60, 61]. Sex differences in reasons for services use could also reflect differences in internalizing and externalizing psychopathology or in the perception of socially acceptable reasons for seeking help [28, 62].

## Limitations and conclusions

Our study is not without limitations. First, self-injury was measured with one item and a limited list of example behaviors that excluded certain self-harming behaviors, such as self-poisoning. More examples of male-typical self-injurious behaviors (e.g., punching a fist into a wall or burning; see [19]) could have resulted in higher rates for males, possibly altering our conclusions about sex differences. Second, self-injury was assessed as self-injurious behavior in the month previous to the assessment. To compare the prevalence with that found in other studies, 6- or 12-month time frames would have been ideal. Moreover, the prevalence estimates likely represent an underestimation of the true prevalence given the 1-month time frame. Third, our self-injury item did not assess the intent of self-injury. However, the examples provided were consistent with NSSI. Fourth, self-injury was assessed beginning at age 13, but the high rates of self-injury at age 13 suggest that this behavior emerges earlier. Childhood self-injury, particularly among boys, may be an important topic for future research. Fifth, our data did not include information on emergency room visits and whether services use was directly associated with prior self-injury. Sixth, it is unclear whether findings from our relatively urban sample can be generalized to other areas of Switzerland and other countries with different socio-economic and cultural contexts and health care systems. Finally, our study was based on adolescent self-reports of self-injury and services use; ideally, these data would be triangulated with administrative or other objective data.

Despite these limitations, our study provides important new insights into the sex-specific age-related course of self-injury from early adolescence to early adulthood. Indeed, it is the first study to document a high prevalence of (recurrent) self-injury in urban Switzerland and to comprehensively examine self-injury prevalence, recurrence, and services use among adolescent males in the community. Future research should examine what factors precipitate the changing rates of self-injury across adolescence, including, for example, changing stressors, stress reactivity, and mental health. Importantly, more work is needed to understand how to better connect adolescents affected by self-injury with appropriate mental health services.

## Electronic supplementary material

Below is the link to the electronic supplementary material.

Supplementary file1 (PDF 228 kb)
