# Supplementary material for: Self-injury from early adolescence to early adulthood: age-related course, recurrence, and services use in males and females from the community
Source: Eur Child Adolesc Psychiatry. 2020 Jun 22;30(6):937–51. doi: 10.1007/s00787-020-01573-w (PMC8140957; doi:10.1007/s00787-020-01573-w)
Supplement: Supplementary file 1 — Supplementary file1 (PDF 228 kb) [file 787_2020_1573_MOESM1_ESM.pdf]

Online Supplement

Self-injury from early adolescence to early adulthood: Age-related course, recurrence, and services use in males and females from the community

Annekatrin Steinhoff, Ph.D., Denis Ribeaud, Ph.D., Stephan Kupferschmid, M.D., Nesrin Raible-Destan, Ph.D., Boris B. Quednow, Ph.D., Urs Hepp, M.D., Manuel Eisner, Ph.D., & Lilly Shanahan, Ph.D.

Corresponding author:

Annekatrin Steinhoff, Jacobs Center for Productive Youth Development, University of Zurich, Andreasstrasse 15, 8050 Zurich, Switzerland. E-Mail: steinhoff@jacobscenter.uzh.ch, phone: +41 44 634 06 92

A) Migration background of participants

Parental immigration background originates in > 80 different countries. Figure A1 shows the seven most frequent countries of birth; several of the countries reflect immigration waves into Switzerland due to specific events such as wars and humanitarian crises.

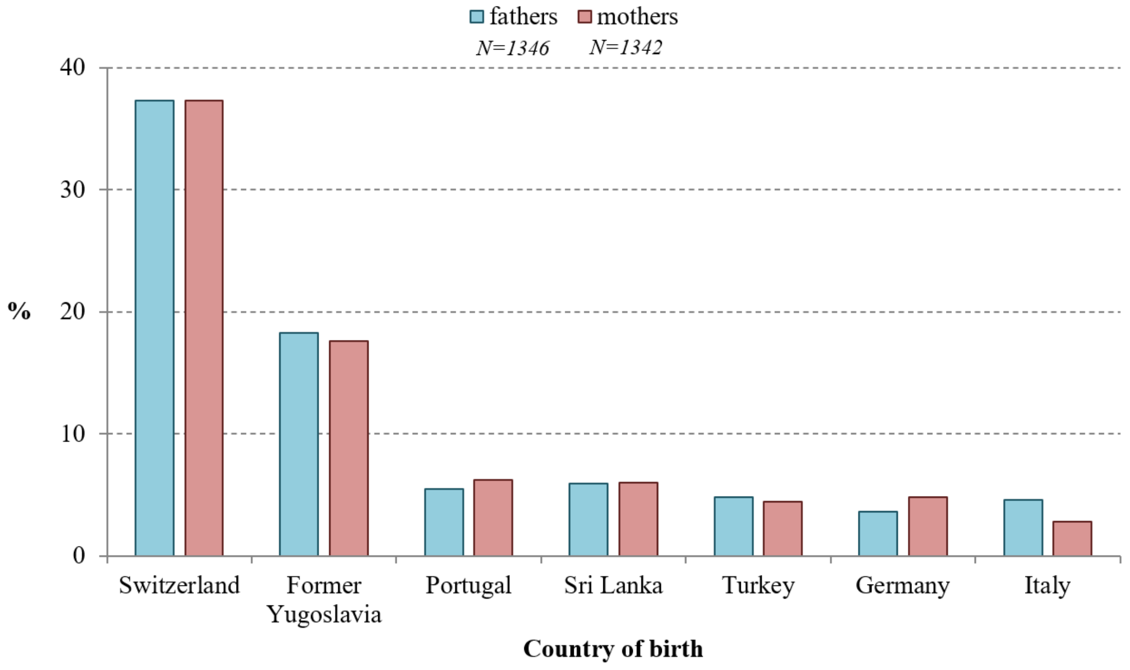

Figure A1. Country of birth of the participants' parents

B) Associations between self-injury and internalizing symptoms and between self-injury and suicidal ideation

Self-injury was associated with internalizing symptoms in our sample (Table B1).

Table B1. Internalizing symptoms mean comparison between adolescents with and without self-injury (ANOVA)

	With self-injury M (SD)	Without self-injury M (SD)	*p* for group difference
**Age 13**	2.81 (0.83)	2.10 (0.66)	< 0.001
**Age 15**	3.06 (0.82)	2.24 (0.72)	< 0.001
**Age 17**	3.30 (0.79)	2.32 (0.75)	< 0.001
**Age 20**	3.45 (0.91)	2.31 (0.75)	< 0.001

Self-injury was also associated with suicidal ideation, which was assessed at ages 15, 17, and 20 (Table B2).

Table B2. Prevalence of suicidal ideations among adolescents with and without self-injury and logistic regression of suicidal ideations on self-injury

	With self-injury (%)	Without self-injury (%)	OR	95% CI	*p*
**Age 15**	55.2	15.1	6.92	4.91–9.75	< 0.001
**Age 17**	68.0	17.6	9.92	6.62–14.87	< 0.001
**Age 20**	63.5	15.8	9.29	5.94–14.51	< 0.001

C) Point prevalence in services use

Overall. We examined both retrospective reports of services use (i.e., reported use of services in the two/three years *before* the interview when self-injury was reported) and prospective rates (i.e., service use *after* the self-injury assessment). The retrospective reports showed that point prevalence of services use in adolescents with self-injury increased from ages 13 to 20 (Figure C1). At each assessment, adolescents with self-injury were more likely to have used services than those without self-injury; this gap widened after mid-adolescence (age 13: OR = 2.02, 95% CI = 1.45–2.80, $p < 0.001$; age 15: OR = 1.99, 95% CI = 1.43–2.78, $p < 0.001$; age 17: OR = 3.88, 95% CI = 2.65–5.67, $p < 0.001$; age 20: OR 3.06, 95% CI = 2.00–4.69, $p < 0.001$). With respect to types of services used, 13- and 15-year-olds with self-injury were most likely to have used school-based services. Beginning at age 17, they more commonly reported having seen clinical psychologists or psychiatrists. Prospective rates of services use (i.e., services use reported two/three years *after* the self-injury assessment) were similar, with the exception of a non-significant difference between youth with and without self-injury at age 17 with regard to school-related services use until age 20.

During late adolescence and early adulthood (i.e., at ages 17 and 20), youth with self-injury were significantly more likely than their peers without self-injury to have been admitted to a psychiatric hospital (13.5% vs. 1.3%, OR = 12.04, 95% CI = 5.85–24.77, $p < 0.001$ at age 17; 24% vs. 2.2%, OR = 13.92, 95% CI = 7.49–25.85, $p < 0.001$ at age 20). Differences in the point prevalence of any hospital admission between those with and those without self-injury were marginally significant at ages 15 and 17 only ($p = 0.18$, $p = 0.067$, $p = 0.050$, $p = 0.26$ for the group difference at ages 13, 15, 17, and 20, respectively, with youth with self-injury being slightly more likely to be hospitalized: OR = 1.49, 95% CI = 0.97–2.29 at age 15; OR = 1.62, 95% CI = 1.00–2.64 at age 17).

Sex differences. Females with self-injury were marginally more likely to have seen a mental health care provider compared to males with self-injury at ages 15 and 20 (Figure C1). At age 17, females with self-injury were more likely than males with self-injury to have seen a clinician. No sex difference emerged in the point prevalence of admission to a psychiatric hospital. However, at age 17, females with current self-injury were more likely than males to have spent several days in hospital (23.6% vs. 5.4%, OR = 5.40, 95% CI = 1.20–24.38, $p = 0.028$).

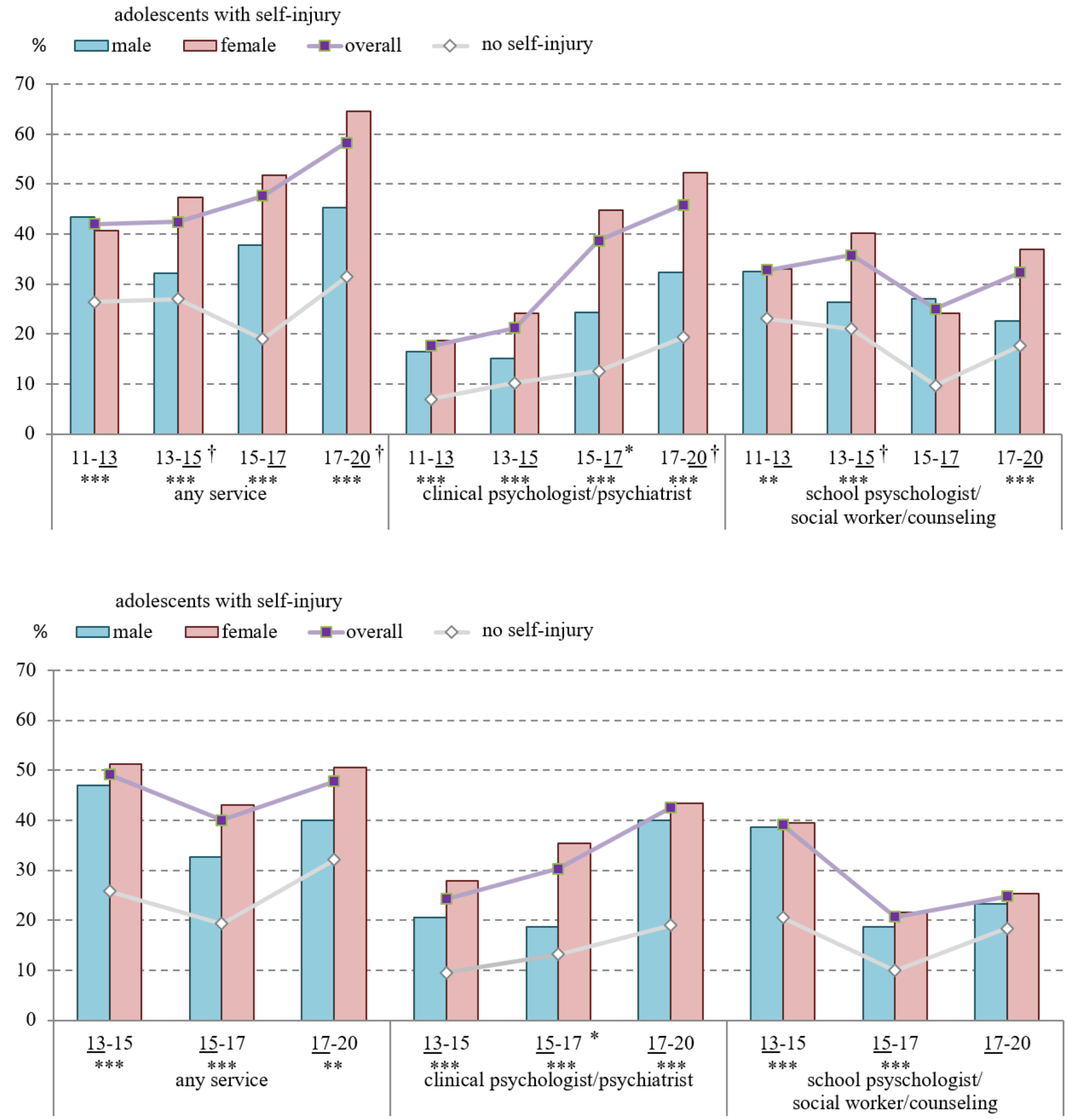

Figure C1. Overall and sex-specific prevalence of services use during the previous two years among adolescents who had or had not self-injured in the previous month.
Top: Retrospective reports (services use during the two years before self-injury assessment)
Bottom: Prospective reports (services use during the two years after self-injury assessment)
Note. Age at self-injury assessment is underlined; *p*-values for sex differences are next to the ages on the x-axis, while *p*-values for differences between adolescents with and without self-injury are below the ages on the x-axis
† $p < 0.10$, * $p < 0.05$, ** $p < 0.01$, *** $p < 0.001$

Point prevalence of reasons for services use

Overall. When examining the point prevalence of reasons for services use among adolescents with self-injury at individual ages, self-harm often did not emerge as the most common reason for having seen a health care provider (Tables C1–C4). At age 13, family problems and attention problems were the most common reasons. From age 15 onwards, depressive symptoms, self-injury, and suicidal thoughts became more common reasons.

Sex differences. During mid-adolescence, females with self-injury reported use of professional services because of depressive symptoms, self-injury, and suicidal thoughts more often than males at the statistical trend level (Tables C1–C4). Males with self-injury at age 13 reported attention deficit problems and learning difficulties as reasons for use of mental health services more often than females with self-injury. With respect to social relationships, family problems were the most salient reasons for seeking help among adolescents with self-injury, particularly females, at all assessments. From mid-adolescence onwards, females with self-injury more commonly reported victimization experiences as a reason for services use than males. In contrast, early adolescent males more often reported using services because they had offended others (e.g., violent offense or bullying).

Table C1. Trends and sex differences in the reasons for services use among adolescents with self-injury at age 13

	**Overall %**	**Male %**	**Female %**	***p* for sex difference**
**Retrospective**	**n = 73**	**n = 36**	**n = 37**	
Depression/self-injury/suicidal thoughts	15.1	< 13.9	21.6	0.11
Attention/concentration problems	19.2	30.6	< 13.5	0.015
Learning difficulties	13.7	22.2	< 13.5	0.037
Substance use	-	-	-	-
Family problems	34.2	27.8	40.5	0.25
Problems with teacher	13.7	13.9	13.5	0.96
Victimization	12.3	< 13.9	16.2	0.31
Perpetration of violence or bullying	8.2	13.9	< 13.5	0.082
**Prospective**	**n = 83**	**n = 39**	**n = 44**	
Depression/self-injury/suicidal thoughts	14.5	< 12.8	22.7	0.023
Attention/concentration problems	15.7	20.5	11.4	0.25
Learning difficulties	13.3	15.4	11.4	0.59
Substance use	8.4	< 12.8	11.4	0.31
Family problems	34.9	25.6	43.2	0.094
Problems with teacher	7.2	12.8	< 11.4	0.064
Victimization	9.6	< 12.8	13.6	0.19
Perpetration of violence or bullying	9.6	15.4	< 11.4	0.095

Note. Retrospective reports relate to services use during the two/three years before the interview when past-month self-injury was assessed, which results in some overlap in timeframes, whereas prospective reports relate to services use during the two/three years after the interview when past-month self-injury was assessed, which does not result in an overlap in timeframes.

Table C2. Trends and sex differences in the reasons for services use among adolescents with self-injury at age 15

	**Overall %**	**Male %**	**Female %**	***p* for sex difference**
**Retrospective**	**n = 70**	**n = 17**	**n = 53**	
Depression/self-injury/suicidal thoughts	21.4	< 29.4	26.4	0.073
Attention/concentration problems	10.0	< 29.4	9.4	0.78
Learning difficulties	10.0	< 29.4	9.4	0.78
Substance use	< 7.1	-	< 9.4	0.42
Family problems	44.3	41.2	45.3	0.77
Problems with teacher	< 7.1	-	< 9.4	0.57
Victimization	12.9	-	17.0	0.069
Perpetration of violence or bullying	8.6	< 29.4	< 9.4	0.59
**Prospective**	**n = 58**	**n = 14**	**n = 44**	
Depression/self-injury/suicidal thoughts	37.9	< 35.7	43.2	0.14
Attention/concentration problems	10.3	< 35.7	< 11.4	0.58
Learning difficulties	13.8	< 35.7	13.6	0.95
Substance use	-	-	-	-
Family problems	34.5	< 35.7	38.6	0.24
Problems with teacher	10.3	< 35.7	< 11.4	0.58
Victimization	13.8	< 35.7	13.6	0.95
Perpetration of violence or bullying	-	-	-	-

Note. Retrospective reports relate to services use during the two/three years before the interview when past-month self-injury was assessed, which results in some overlap in timeframes, whereas prospective reports relate to services use during the two/three years after the interview when past-month self-injury was assessed, which does not result in an overlap in timeframes.

Table C3. Trends and sex differences in the reasons for services use among adolescents with self-injury at age 17

	**Overall %**	**Male %**	**Female %**	***p* for sex difference**
**Retrospective**	**n = 59**	**n = 14**	**n = 45**	
Depression/self-injury/suicidal thoughts	44.1	< 35.7	51.1	0.051
Attention/concentration problems	13.6	< 35.7	15.6	0.42
Learning difficulties	16.9	< 35.7	20.0	0.26
Substance use	< 8.5	< 35.7	< 11.1	0.073
Family problems	23.7	-	31.1	0.017
Problems with teacher	10.2	< 35.7	< 11.1	0.56
Victimization	10.2	-	13.3	0.15
Perpetration of violence or bullying	< 8.5	< 35.7	-	0.071
**Prospective**	**n = 54**	**n = 12**	**n = 42**	
Depression/self-injury/suicidal thoughts	63.0	50.0	66.7	0.29
Attention/concentration problems	14.8	< 41.7	11.9	0.26
Learning difficulties	14.8	< 41.7	14.3	0.84
Substance use	9.3	< 41.7	< 11.9	0.32
Family problems	27.8	< 41.7	31.0	0.33
Problems with teacher	< 9.3	-	< 11.9	0.44
Victimization	< 9.3	-	< 11.9	0.27
Perpetration of violence or bullying	-	-	-	-

Note. Retrospective reports relate to services use during the two/three years before the interview when past-month self-injury was assessed, which results in some overlap in timeframes, whereas prospective reports relate to services use during the two/three years after the interview when past-month self-injury was assessed, which does not result in an overlap in timeframes.

Table C4. Trends and sex differences in the reasons for services use among adolescents with self-injury at age 20

	**Overall %**	**Male %**	**Female %**	***p* for sex difference**
**Retrospective**	**n = 56**	**n = 14**	**n = 42**	
Depression/self-injury/suicidal thoughts	57.1	50.0.0	59.5	0.53
Attention/concentration problems	16.1	< 35.7	16.7	0.83
Learning difficulties	16.1	< 35.7	14.3	0.53
Substance use	< 8.9	< 35.7	< 11.9	> 0.99
Family problems	32.1	< 35.7	38.1	0.099
Problems with teacher	< 8.9	< 35.7	< 11.9	> 0.99
Victimization	17.9	< 35.7	21.4	0.23
Perpetration of violence of bullying	< 8.9	< 35.7	< 11.9	0.41

Note. Retrospective reports relate to services use during the two/three years before the interview when past-month self-injury was assessed, resulting in so some overlap in timeframes.
